# Development and evaluation of a massive open online course (MOOC) to teach medical students the prudent use of antibiotics

**DOI:** 10.1186/s13756-025-01640-4

**Published:** 2025-09-22

**Authors:** Miriam Wiese-Posselt, Selin Saydan, Thiên-Trí Lâm, Alina Rörig, Clara Bergmann, Felicia Becker, Oliver Kurzai, Markus A. Feufel, Petra Gastmeier, Sandra Schneider

**Affiliations:** 1 Institute of Hygiene and Environmental Medicine, Charité - Universitätsmedizin Berlin Corporate Member of Freie Universität Berlin, Humboldt-Universität zu Berlin, Berlin, Germany; 2 Institute for Hygiene and Microbiology, University of Würzburg, Würzburg, Germany; 3 Department of Psychology and Ergonomics (IPA), Division of Ergonomics, Technische Universität Berlin, Berlin, Germany; 4 Helios Klinikum Emil-von-Behring, Walterhöfer Str. 11, 14165 Berlin, Germany

**Keywords:** Antibiotic use, Medical students, Undergraduate education, Massive open online course (MOOC), Antimicrobial resistance, FOAM (free open access medical education)

## Abstract

**Background:**

If antibiotics are used appropriately, the development of antimicrobial resistance (AMR) can be curbed. Many medical students feel that they do not receive sufficient training in this respect during their undergraduate medical education. In recent years, digital learning formats are being successfully employed in student teaching. Our aim was to develop and evaluate a massive open online course (MOOC) on appropriate antibiotic therapy and the development of AMR. The intention was to provide the MOOC as an effective learning format in medical schools and to encourage others to develop their own MOOCs on other topics.

**Methods:**

We developed a MOOC for medical students that consisted of four modules (M1-4) on bacteriology, microbiology diagnostics, pharmacology, antibiotics, AMR, the One Health approach, principles of appropriate antibiotic therapy, and transfer of knowledge to clinical practice. MOOC learners were asked to answer the same 16 knowledge and five self-assessment questions at the beginning and end of the MOOC and to give course feedback in an anonymous online questionnaire.

**Results:**

From July 1, 2021 until June 30, 2022 the MOOC was actively attended by 2061 learners. Of them, 473 (23%) completed the final exam and 389 (19%) answered the knowledge and self-assessment questions at the beginning and end of the MOOC. A significant increase in knowledge and a strengthening of competence and self-confidence was observed in these 389 learners. The median knowledge score increased significantly from 10/16 (IQR 8; 12) points before the MOOC to 16/16 (15; 16) afterwards (*p* < 0.001). Overall, course activity decreased from M1 (100% attendance) to M4 (38%). At the end of the MOOC, the online feedback questionnaire was completed by 304 (15%) learners, most of whom rated the MOOC positively. For example, 97% of them stated that they had increased their knowledge in relevant areas.

**Conclusions:**

A high dropout rate for voluntary MOOCs is generally reported. Therefore, a course completion rate of 23% is acceptable. The learners who completed the MOOC showed a significant increase in knowledge and self-confidence. The use of the MOOC, or parts of it, as digital learning format for undergraduate medical education appears promising.

**Supplementary Information:**

The online version contains supplementary material available at 10.1186/s13756-025-01640-4.

## Introduction

The inappropriate use of antibiotics is one of the main drivers in the development of antimicrobial resistance (AMR) [1, 2]. Medical students are an important target group to address this topic. Several surveys among medical students showed that they are aware of their crucial role as future prescribers. However, gaps in knowledge and a lack of competence in the appropriate use of antibiotics could be identified even among students in higher semesters [3–8]. In several surveys on attitudes and perceptions concerning their training in antibiotics, medical students seemed to prefer easy access teaching with a constant reference to clinical relevance. They also stated that they appreciate teaching with regular feedback and checks of what they had learned as well as interaction with peers and teachers/experts [3, 7, 8]. In a focus group discussion with German medical students in one of our own projects, we learned that they consider basics of bacteriology and microbiological diagnostics and a profound pharmacological knowledge of antibiotic drugs as important areas of knowledge. But only if these fields can be linked to clinical practice right from the start [7]. In recent years, e-learning formats have become a popular teaching approach for medical students, for example through the use of apps, online courses or gaming [9–13]. Beginning in March 2020, we all were faced with repeated lockdowns and the closure of universities due to the SARS-CoV-2 pandemic. Consequently, online teaching became an essential tool for education and training [14]. Online learning formats provide low-threshold access; they can be used from anywhere and at any time. They also offer students the opportunity to develop their own learning styles and time management skills [15, 16]. With this in consideration, we developed a target group tailored online course for medical students that was intended to foster knowledge and competences on the prudent use of antibiotics. Based on previous experience with a format that is very adaptable, we decided in favour of a massive open online course (MOOC) [17]. The aim of this article is to assess the impact of this MOOC on the learners’ knowledge gain, competence and self-confidence, and to present their feedback on the learning tool. We also describe the structure, content and delivery of the MOOC. By doing so, we intent to promote the use of this MOOC or parts of it in other medical faculties or to encourage others to develop their own online format based on our blueprint.

This work was carried out as part of the network project RAI (Responsible antibiotic use via information and communication) targeting medical students (InfectControl) [18].

## Methods

The composition of the MOOC content was discussed and agreed upon by the physicians and researchers in our project group. We also involved medical students in launching, promoting and running the course. The MOOC was in German and consisted of four modules (M).

- M1 – focus on bacteria: covered basic bacteriology, e.g., common Gram-positive and Gram-negative infectious bacteria as well as the basics of microbiological diagnostics from the preanalytical phase to the interpretation of bacteriological findings.
- M2 – focus on antibacterials: dealt with the basics of antibiotic substances, including the main mechanisms of action of the different antibiotic classes as well as the pathogen spectrum and important pharmacological aspects of clinically relevant antibiotics.
- M3 – focus on epidemiology: covered the topics AMR development, multi-resistant pathogens and their epidemiology, principles of appropriate antibiotic therapy, and the One Health approach.
- M4 – focus on clinical application: all the prior information was applied to the following clinical areas and clinical cases: respiratory infections in primary care, important bacterial infections in internal medicine, urinary tract infections, perioperative antibiotic prophylaxis, and antibiotic use in intensive care.

MOOC learners were free to choose which modules to work on. That meant they could skip learning units in one module or entire modules and move on to the next module if they felt they had already received sufficient training in the respective topic. They were asked to complete the MOOC with an exam within three months if possible. However, the exam could also be taken later up to the end of the course on June 30, 2022. Each module included five learning videos (6–23 min in length) provided by medical lecturers from the fields of microbiology, pharmacy, infection prevention and control, infectious diseases, and antibiotic stewardship. The learning videos were based on currently published guidelines, data, and articles. Additional learning materials, such as references to guidelines, websites, and professional publications, were provided in the MOOC. It also offered other digital learning formats, including explanatory films, podcast episodes and interactive clinical cases. Short series of learning videos were followed by multiple-choice questions (MCQs) for self-assessment. The learners received feedback on their answers. With the help of the provided materials, they were able to fill in gaps in their knowledge. In addition, explanations were provided for the most complex MCQs. There was also a discussion forum in the MOOC where learners could interact and/or exchange ideas with MOOC tutors. Moreover, they were able to contact tutors via email as well. MOOC users were invited to complete the online course by taking an exam consisting of two parts with 40 MCQs each. To pass the exam and receive a course certificate, at least 75% of the questions had to be answered correctly. You can find detailed information on the structure and content of the MOOC and the references used by the lecturers to prepare the learning videos in the Additional file 1. The learning videos were recorded in a professional studio at the Hasso-Plattner-Institute (HPI), Potsdam, Germany. They show both the lecturer and their power point presentation. The presentation files could be downloaded from the course platform. The MOOC was provided free of charge on HPI’s mooc.house platform after initial registration.

On July 1, 2021, we opened the MOOC for registration. From October 1, 2021 to June 30, 2022, the MOOC was fully available and could be completed along with the exam. We promoted the MOOC throughout Germany and German-speaking Europe. Medical students were informed about the MOOC frequently via their student councils and invited to participate. In addition, the MOOC was presented to department chairs and lecturers in microbiology, infection prevention and control, general medicine, and infectious diseases. We used the following methods to evaluate the learning success, the increase in self-confidence and the user feedback from the course:

- 16 MCQs on course content of all four modules. The same MCQs were provided at the beginning of the MOOC and integrated into the exam at the end of the course. We assessed knowledge gain by calculating the frequency of correct answers.
- Five self-assessment questions on the appropriate use of antibiotics for common infections. The same five questions were asked at the beginning and the end of the MOOC. We evaluated self-assessments by comparing the answers given at the specific times during the course.
- Feedback on the MOOC by means of anonymous data collection in an online questionnaire using LimeSurvey software to evaluate the overall course, the content of the individual modules, and the effectiveness of the learning materials or formats implemented.

HPI provided user data and the results of the knowledge and self-assessment questions. Feedback data was accessed via the LimeSurvey account. Descriptive data analysis was performed using R (https://www.r-project.org/). Number and percent, median, or minimum, interquartile range, and maximum were calculated for the descriptive analysis. The differences were tested using either the chi-squared test, the McNemar test, or the Wilcoxon rank-sum test.

The HPI’s mooc.house platform is no longer in use. Therefore, we transferred the MOOC after the study period to Charité’s Moodle platform (https://lehre.charite.de/). You can access it free of charge after registration by emailing to rai-info@charite.de with the subject “moodle”. Our project website https://www.rai-projekt.de/students provides three exemplary videos of the MOOC with free access.

## Results

### Course user behaviour

**Fig. 1:**
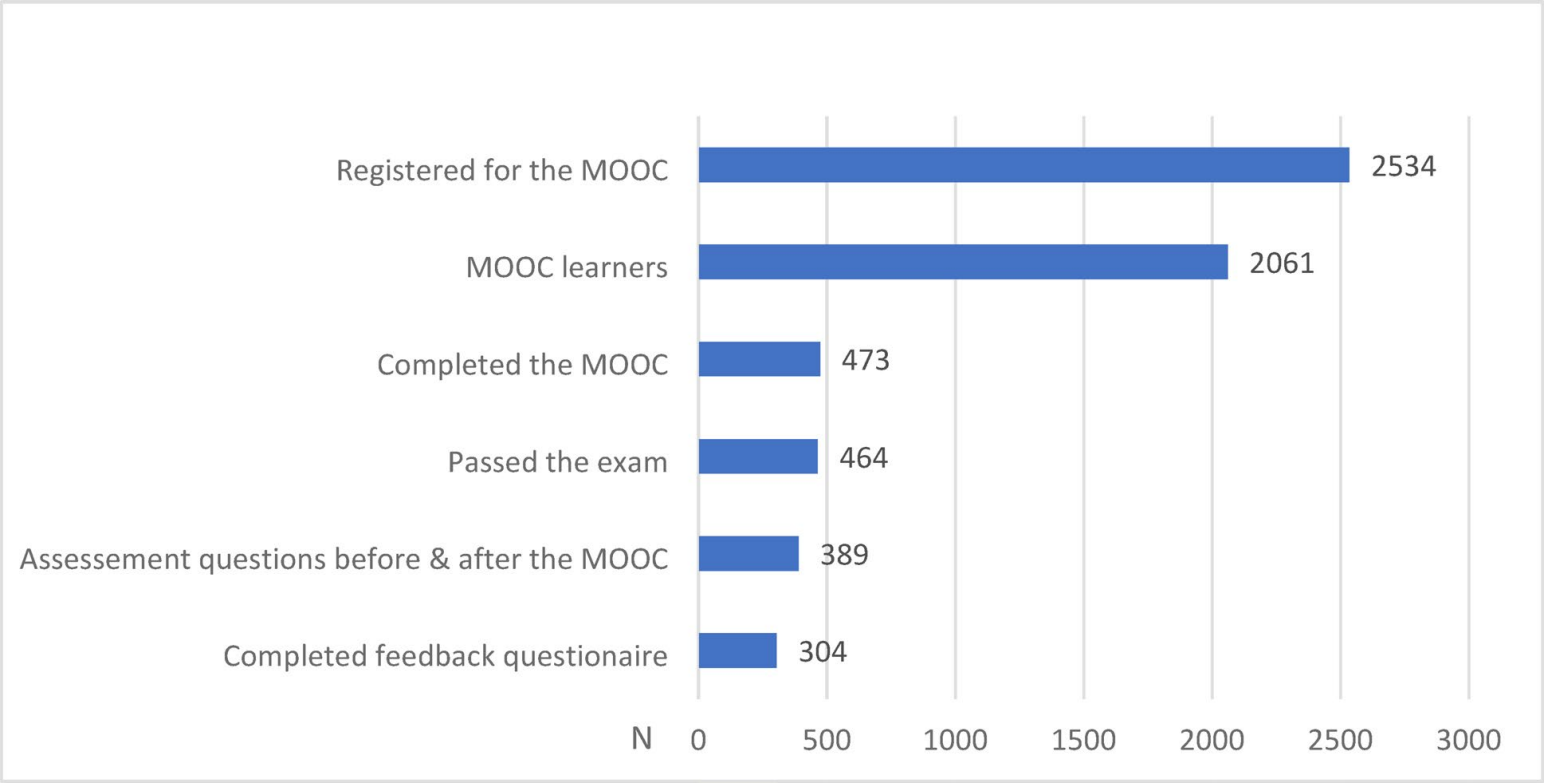
MOOC using behaviour based on the 2534 registered individuals. MOOC term 2021–2022

During the one-year study period the MOOC had 2534 registrations. 2061 individuals actively participated in the course. That is, these persons attended at least one of the MOOC’s learning units at any of the four modules. Figure 1 presents an overview of the participation in the MOOC. The majority (88%) of the 2061 MOOC learners logged in from Germany, 2.8% from Austria, and 2.3% from Switzerland; the remaining 7% logged in from other European countries or from outside Europe (e.g. from the USA).

**Fig. 2:**
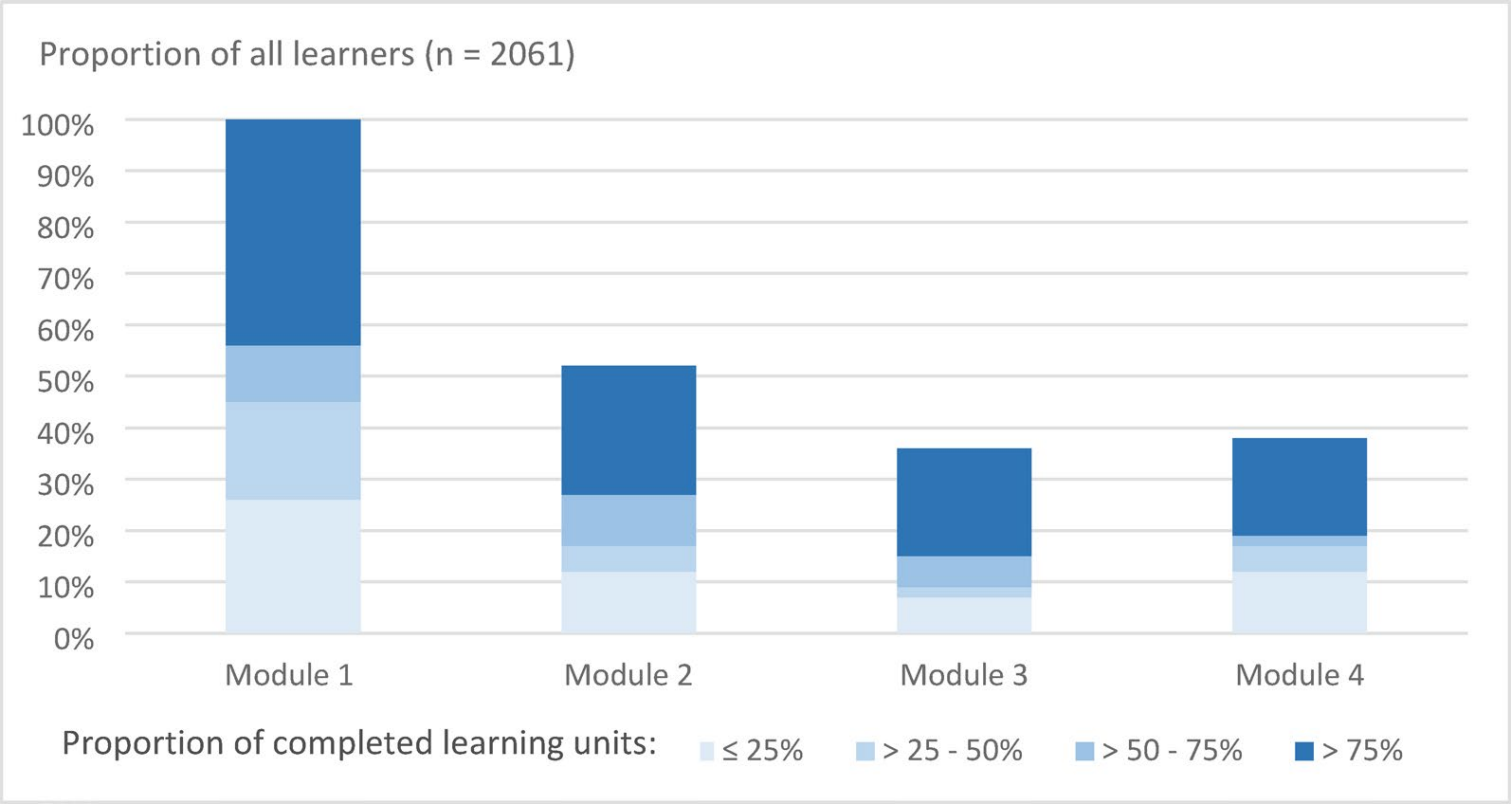
Overview of usage of the MOOC. Proportion of learners who accessed the individual modules, as well as proportion of learning units completed in each module, divided into four categories: completion of ≤ 25% of the learning units, > 25–50%, > 50–75%, or > 75%. The proportions of participants attending per module are reflected in the total columns. MOOC term 2021–2022

The number of MOOC learners varied between the four modules, with the highest number of participants in M1 and the lowest in M3. M1 was attended by almost 100% of the learners (*n* = 2059), M2 by 1077 learners (52%), M3 by 720 (35%), and M4 by 775 (38%). Figure 2 gives an overview of the completed learning units by module.

The self-assessment quizzes were completed by 870 (42%) of 2061 MOOC learners in M1, 592 (29%) learners in M2, 441 (22%) learners in M3 and 430 (21%) learners in M4, respectively. Of the 2061 learners, 473 (23%) completed the exam, which covered the content of M1 to M4 and was based on 80 MCQs. Of these, 464 (96%) answered at least 75% of all the questions correctly, and thus successfully passed the exam. During the MOOC, 984 (48%) learners visited the discussion forum and 13 posted a total of 19 contributions there. Over the course of the MOOC, 51 learners contacted MOOC tutors via email. These messages focused on organisational aspects and questions about the functionality of the MOOC.

### Gaining knowledge and self-confidence

To estimate learning success, we looked at the learners who had answered both the 16 knowledge MCQs and the five self-assessment questions at the beginning and end of the MOOC. This data was available for 389 learners (19% of all learners). These 389 participants were significantly more active than others in the MOOC (see Table S1 in the Additional file 2). The percentage of correct answers given by the 389 learners increased significantly for all 16 knowledge MCQs after their participation in the MOOC. Awarding one point for each correct answer, the median knowledge score increased from 10 (IQR 8; 12) points at the beginning of the MOOC to 16 (IQR 15; 16) after its completion (Wilcoxon rank-sum test *p* < 0.001), with 16 being the highest achievable score. Table 1 shows the percentage of correct responses to the 16 knowledge MCQs at the beginning and the end of the MOOC. In addition, the degree of knowledge gained for each question is reported. The highest percentage increase in knowledge was 61%, the lowest 7%. The complete data set on the 16 knowledge MCQs and the answers of the 389 MOOC learners can be found in Table S2 in the Additional file 2.

**Table 1:** Number, percentage of correct answers and increase in correct answers to the 16 knowledge MCQs at the beginning of the MOOC and in the exam at the end of the course. The questions are sorted in the tabular overview according to the degree of increase in correct answers; *n* = 389 MOOC learners, MOOC term 2021–2022. (See also table S2)

	Correct answers of the MOOC learners (*n* = 389) tabulated in order of gain in learning success			
	At the beginning of the MOOC^a^	At the end of the MOOC (in the exam)^a^	Gain in correct answers^a^	*p*-value^b^
*Questions*				
Which is not part of the preanalytics?	357 (92%)	385 (99%)	+ 28 (7%)	< 0.001
What are the most common pathogens of community-acquired pneumonia (CAP)?	348 (89%)	386 (99%)	+ 38 (10%)	< 0.001
Which statement best describes the selection of antibiotic resistance?	324 (83%)	376 (97%)	+ 52 (14%)	< 0.001
Which abbreviation is correct (note: regarding AMR, common in Germany)?	305 (78%)	382 (98%)	+ 77 (20%)	< 0.001
Which statement is true (note: regarding diagnosis and therapy of acute urinary tract infections)?	273 (70%)	362 (93%)	+ 89 (23%)	< 0.001
Which antibiotic belongs to the beta-lactam antibiotics?	291 (75%)	385 (99%)	+ 94 (24%)	< 0.001
Which antibiotic is the first choice for mild CAP without comorbidities?	283 (73%)	381 (98%)	+ 98 (25%)	< 0.001
Which statement about the principles of prudent antibiotic therapy is true?	278 (71%)	379 (97%)	+ 101 (26%)	< 0.001
Which of the following pathogens does not belong to the enterobacteriaceae?	251 (65%)	363 (93%)	+ 112 (28%)	< 0.001
How do fluoroquinolones work?	251 (65%)	379 (97%)	+ 128 (32%)	< 0.001
Which bacteria are not Gram-positive?	217 (56%)	371 (95%)	+ 154 (39%)	< 0.001
Which of the following agents is the first-line treatment for proven methicillin-sensitive *Staphylococcus aureus*?	213 (55%)	375 (96%)	+ 162 (41%)	< 0.001
Acute bronchitis: What percentage of illnesses are viral?	216 (56%)	384 (99%)	+ 168 (43%)	< 0.001
CAP: Which of the following statements is true (note: regarding diagnostics and therapy)?	100 (26%)	293 (75%)	+ 193 (49%)	< 0.001
Acute bronchitis: Which of the following statements is true (note: regarding symptoms and differential diagnosis)?	149 (38%)	382 (98%)	+ 233 (60%)	< 0.001
The approximate proportion of carbapenem-resistant *Klebsiella pneumoniae* strains in India is?	124 (32%)	361 (93%)	+ 237 (61%)	< 0.001

^a^ Given in number and percentage n (%)

^b^ McNemar-Test

**Fig. 3:**
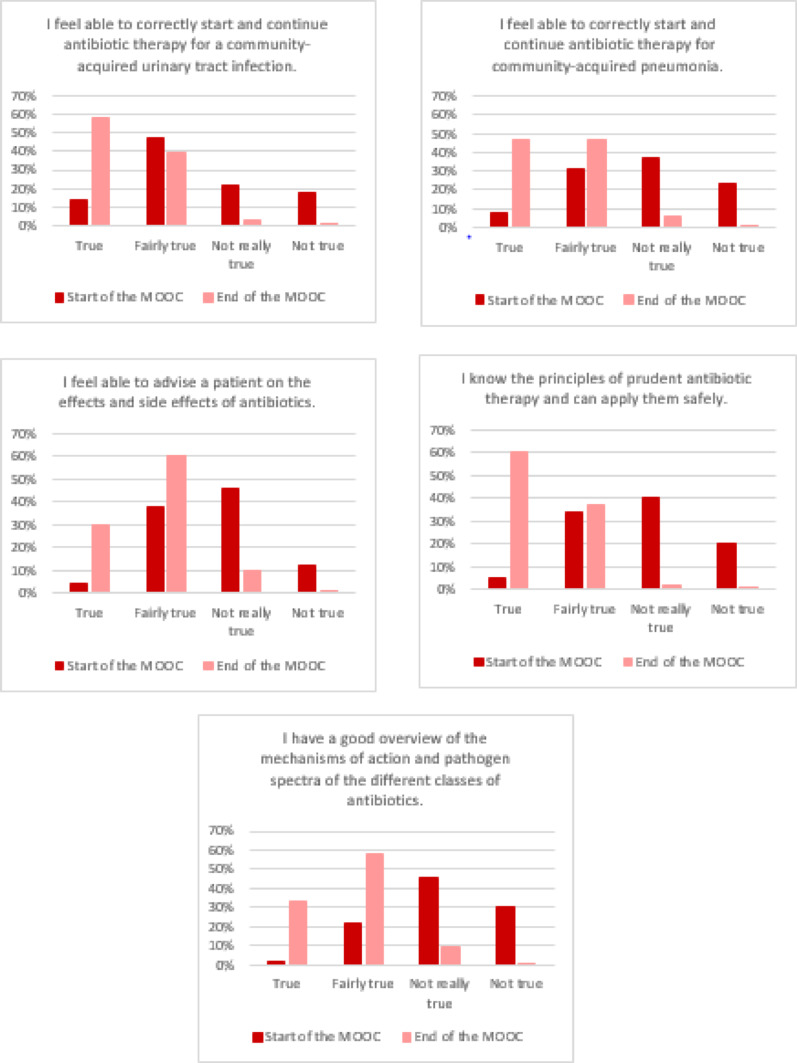
Overview of the self-assessment questions of MOOC learners (*n* = 389) on confidence in their abilities regarding appropriate antibiotic therapy; MOOC term 2021–2022

The results of the five self-assessment questions showed that the 389 MOOC learners, who answered the self-assessment questions both before and after the course, were significantly more confident in their ability to provide appropriate antibiotic therapy following active participation in the course. After the MOOC, they felt more confident in their knowledge of the principles of appropriate antibiotic use and in counselling patients (Fig. 3). For example, 225 of 389 (58%) learners rated the statement “I feel able to correctly start and continue antibiotic therapy for a community-acquired urinary tract infection.” as true for themselves after attending the MOOC, compared to only 54 (14%) before the course. This increase in self-confidence was significant for all five statements (*p* < 0.001), see Fig. 3.

### Course feedback

We received 304 completed feedback questionnaires. This refers to 15% of all MOOC learners (*n* = 2061) and 64% of those who completed the course (*n* = 473). Most of the feedback providers (283 (93%)) were medical students and 189 (62%) were female. The median age group was 23–25 years of age. Of the 283 medical students, 216 (76%) were in advanced semesters (7th to 12th semester). Physicians at the start of their careers, pharmacists, and natural scientists were among the respondents who were not studying medicine at the time.

**Fig. 4:**
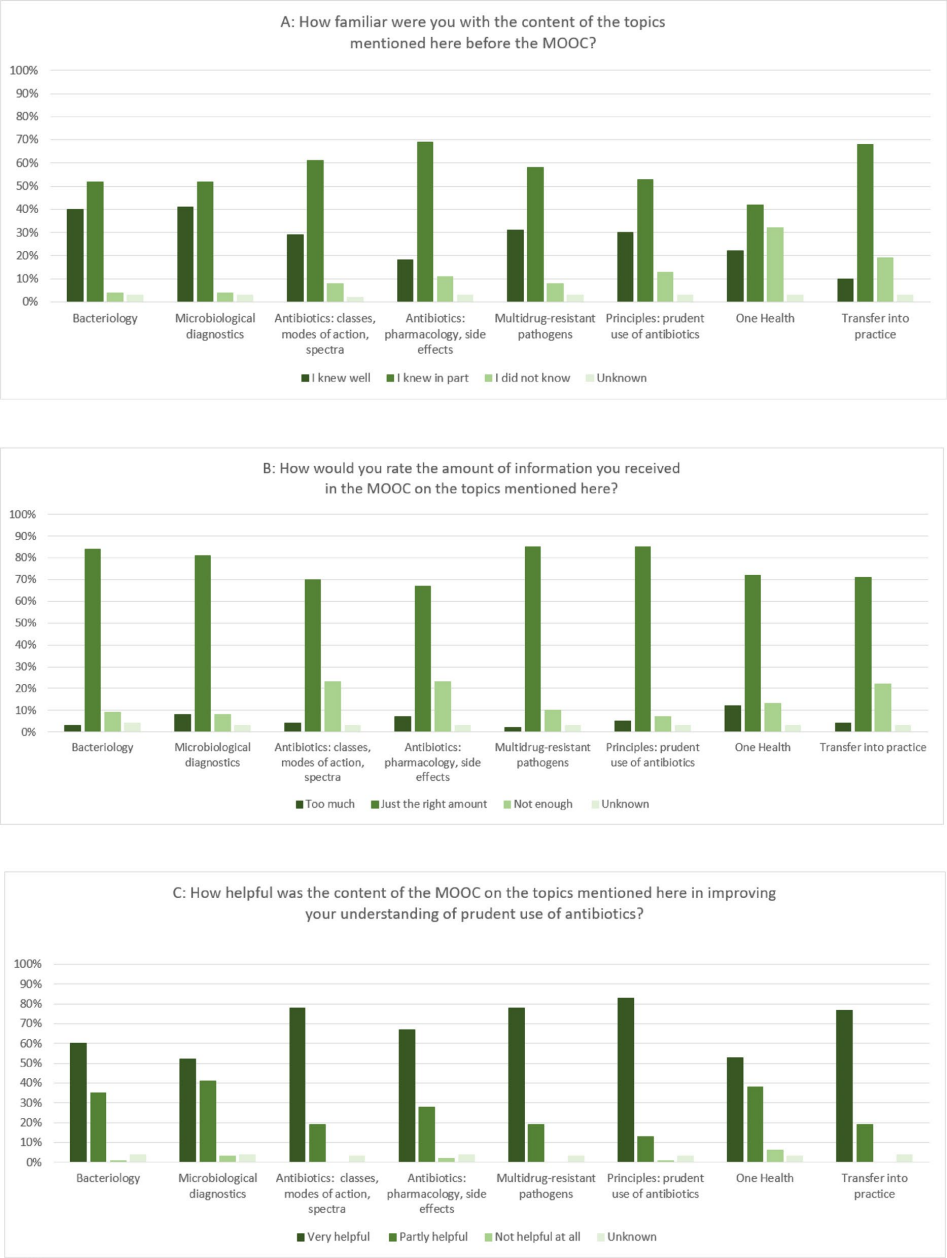
Feedback on the MOOC in terms of (4 A) the level of prior knowledge, (4B) the amount of information provided in the MOOC, and (4 C) the relevance of the course content for understanding the aspects of prudent antibiotic use; *n* = 304 feedback giver, MOOC term 2021–2022

Overall, the MOOC was rated very positively in terms of the knowledge acquired and the increased self-confidence in the appropriate use of antibiotics. The feedback providers reported that they had a better understanding of the links between the use of antibiotics and the development of AMR after participating in the MOOC. In addition, they saw the benefit of this knowledge for their current medical training and for their future work as doctors (see Table S3 in the Additional file 2). The information on learners’ knowledge prior to the MOOC and their assessment of the scope of the course content and the impact of the acquired knowledge on their understanding of prudent antibiotic use is presented in Fig. 4.

With regard to the learning content of M1, 122 (40%) of the 304 learners who responded to the feedback survey stated that they had knowledge of bacteriology before participating in the MOOC. In the other modules, however, there were larger gaps in the knowledge participants had prior to taking the MOOC: for example, only 54 (18%) feedback survey respondents stated that they were very familiar with the pharmacological aspects and side effects of antibiotics (M2) and only 68 (22%) knew anything about the topic One Health (M3). Despite these differences in knowledge prior to the MOOC, the majority of feedback givers judged the amount of information in the four modules to be generally “just the right amount” (67–85%). With regard to the appropriate use of antibiotics, over 90% of participants in the feedback survey rated the knowledge imparted in the individual modules of the MOOC as “very helpful” or “partially helpful” (Fig. 4). Some of those who provided feedback made free comments. For example, some wanted more learning videos on other topics and more opportunities to interact with the lecturers.

## Discussion

Our goal was to provide medical students with a target group-tailored, easily accessible learning format that increases the knowledge and competences of the learners in order to support the appropriate use of antibiotics. We report here on the successful development and delivery of a MOOC on this topic for undergraduates in medical school. In recent years, several articles on MOOCs related to antibiotic stewardship (ABS) have been published. However, most of these MOOCs are aimed at healthcare professionals rather than medical students [13, 17, 19–21]. Moreover, they focus on ABS and place less emphasis on foundational topics such as pharmacology or microbiology related to the appropriate antibiotic use, which were central components of our MOOC. MOOCs on ABS are also available on the Internet, e.g. from Stanford University (edX platform) or from the WHO, which offers MOOCs worldwide on its own platform [22, 23]. While some MOOCs are freely available, access to certain platforms requires payment or a subscription. Mostly, these MOOCs require prior experience in the field [22–25]. However, Malli et al. reported that the WHO-ABS MOOC, that had been offered by https://openwho.org/, is considered a valuable resource for use in formative medical curricula [26]. Since a few years, medical students are already accustomed to online teaching. MOOCs and locally organized online courses offer a wide range of digital learning formats – including learning videos, simulations, and clinical case scenarios – and are widely available, both commercially and free of charge. Students emphasize that an effective MOOC must be of high quality, which necessitates the development of a centralized curriculum and the creation of content specifically tailored to the needs of medical students [27]. We specifically designed our MOOC for medical students at German universities, aligning the course content with the learning objectives defined in the National Competence-Based Catalogue of Learning Objectives for Undergraduate Medical Education (NKLM) [28]. The video-based learning units were delivered by faculty members from our medical school, who were also available for student support throughout the course via discussion forums or email. This approach distinguished our MOOC from large-scale courses offered on platforms such as edX or by the WHO, as well as from MOOCs designed primarily for healthcare professionals. In the following section, we compare the performance of our MOOC with those of local online courses and other MOOCs covering different topics in undergraduate medical education such as oncology, anatomy, occupational medicine, and transplant medicine. These courses were consistently evaluated positively by students, particularly when the learning format supplemented on-campus teaching or was integrated into a blended learning approach [29–35]. Furthermore, a significant increase in knowledge was demonstrated following participation in a MOOC or an elective online course [11, 26, 30, 33–35].

### Knowledge gained and course feedback

The analysis of responses to identical knowledge MCQs and self-assessment questions at the beginning and end of the course by 389 learners of our MOOC showed a significant increase in knowledge (Table 1). In addition, learners reported increased confidence in appropriately using antibiotics in their clinical practice after completing the MOOC (Fig. 3). This means that we have achieved the goal of imparting knowledge and competences through the active participation in our MOOC. Our findings are in line with reports from other MOOCs or online courses developed for medical students [30, 33–35]. For example, participation in a MOOC on mechanical ventilation resulted in a significant improvement in test scores, increasing from a baseline of 77.5 to 83.1 out of 100 (*p* < 0.001) [33]. In our MOOC, the median knowledge score increased significantly from 10/16 (IQR 8; 12) points before the MOOC to 16/16 (15; 16) afterwards (*p* < 0.001).

Overall, the feedback on our MOOC was highly positive. However, some participants perceived certain MOOC topics as not relevant to their individual learning needs. To address this variability, the implementation of personalized learning pathways appears to be a promising approach as described by Ibrahim et al. in the creating of a MOOC on injury prevention education [36]. Depending on the learner’s degree of autonomy and self-directed learning behavior, the course structure should be designed to foster engagement and sustain learning motivation [31]. Accordingly, participants of our MOOC had the possibility to freely choose which learning formats and content to engage based on their individual preferences.

Based on the results of the course feedback, we were able to identify points that we feel could be improved. It has been reported that the ideal length of learning videos is between 6 and 10 min [37, 38]. Since some of our videos are even longer than 20 min, we would like to break up the content of individual videos into several shorter ones. Furthermore, we would like to follow the advice of some feedback providers and promote greater interaction between MOOC learners and lecturers. Live webinars or interviews with experts, for example, could be used [32, 34]. Or we could encourage participation in the discussion forum through regular communication with learners via social media or appropriate channels in the MOOC [15].

### Challenges in running the MOOC

One of the major disadvantages of MOOCs is a high dropout rate [37, 39]. For example, a dropout rate of 90% was reported by an interview study with MOOC learners [39]. The analysis of the user data of our MOOC showed that of the 2534 registered individuals, 2061 (81%) actively participated in the MOOC as learners. Of these active learners, 473 (23%) completed the final exam, which may be considered an acceptable completion rate. In comparison, in the MOOC studied by Sneddon et al., 11,661 (35%) of the 32,944 registered participants remained active learners. Of these, 663 (6%) completed the course after week 5 by submitting an assignment [13]. In our MOOC, we observed a high dropout rate after M1 (see Fig. 2); thus, between 48% and 65% of MOOC learners did not attend subsequent modules M2-4. We did not record the reasons for leaving the course. But perhaps a part of the MOOC learners was particularly interested in the basic topics of M1. Due to the reforms to the medical degree program in Germany with the aim of better dovetailing practical-clinical and basic science content, it was reported that less content of the basic subjects, such as microbiology, was being taught [40]. The need for teaching in basic topics, such as microbiology or pharmacology, had already been expressed by medical students in focus group discussions that we held before developing the digital learning formats [7]. We believe that our MOOC has the advantage that, on the one hand, the basic knowledge is immediately put into clinical application within the same course and, on the other hand, it is possible to only deepen the basics in a targeted manner if this is required. MOOC learners had the possibility to skip individual learning units or entire modules if they thought they already knew the content of a unit or module. As already mentioned, we plan to strengthen this approach and create individual learning pathways based on the learners’ prior knowledge and competencies before starting the MOOC. With this, and by increasing interaction with learners, we aim to improve the completion rate of our MOOC.

It has been reported that, in addition to the learner’s self-motivation, the course design and content, a lack of available time is one of the main factors contributing to MOOC dropout [39]. Evidence suggests that, for undergraduate learners, the integration of digital learning formats, such as MOOCs, into blended learning settings or flipped classroom models is received very positively and leads to measurable knowledge gains [41–44]. With the adoption of Moodle as the Charité’s learning management system, practical opportunities for implementing blended learning and the flipped classroom concept have become readily available. Due to the flexible structure of our MOOC, both the full course and individual elements can be integrated into these modern teaching strategies.

Technical imponderables or a language barrier might also be causes for high dropout rates [37]. The functionality as well as the structure of our MOOC were rated very positively. Because we designed our MOOC for German-speaking regions, language barriers did not play a role in its evaluation. However, it might be worthwhile to also offer the MOOC in English so that it would be accessible to international students. Another reason for high dropout rates is seen in the voluntary nature of most MOOCs. For example, at the two universities in Paris, an oncology MOOC was set up for medical students as part of the curriculum and was mandatory at one of the universities. The completion rate for the course at the Parisian universities was 70% [30]. Mandatory participation apparently led to a high completion rate in this course.

### Learning tools within the MOOC

In our MOOC, we provided learning videos from medical lecturers. In addition, learners were able to repeat and internalise content learned by taking self-tests (quizzes) at the end of each topic as well as by checking their learning success in the final exam. This video and self-test structure for repetition was well accepted. During the run of the course we also integrated our other learning formats from the RAI-students-project (explanatory films, podcast episodes and digital patient case scenarios) as additional tools in the course of the MOOC [45]. It appears that the additional learning formats did not provide any further benefit to the learners within the MOOC structure but worked better on their own and with partly distinct target groups [45].

A growing body of digital learning tools has been described in the context of undergraduate medical education, including gaming apps, online (live) simulations, interactive case scenarios, and more [12, 29, 34, 46, 47]. Studies have demonstrated that each digital learning format can serve as an effective tool for knowledge transfer, occasionally using playful elements. However, considerable didactic expertise is likely needed to create meaningful learning units that leverage these tools’ strengths without overwhelming students or making the content overly complex.

### Ongoing development of our MOOC

Based on the discussed considerations and on the evaluation of our MOOC, we are pleased to continue offering it as an open-access, free online course for undergraduate medical education. Furthermore, the individual learning components – such as learning videos, quizzes, and explanatory films – are utilized and made available within blended learning frameworks and other digital educational tools. As already mentioned, the MOOC was transferred from the HPI’s platform to Charité’s Moodle platform in beginning of 2023 (https://lehre.charite.de/). Since then, we have been offering Charité medical students the complete MOOC as an optional learning resource on the Moodle platform. In two and a half years, 750 learners registered for the MOOC, of whom 151 (20%) completed it with the exam. Students and interested parties from other universities can also access this MOOC by registering via email at rai-info@charite.de. We are currently revising several learning videos and conducting a comprehensive review of all course materials to ensure their up-to-dateness and quality. Furthermore, we plan to implement personalized learning paths in Moodle, ideally making use of advanced features such as the competency framework, tailored learning plans, and specialized plugins. Moreover, our medical faculty runs a peer teaching program. As a blended learning approach, we have integrated the content and some learning videos and quizzes (MCQs) of the MOOC for a course on the practical use of antibiotics into this program. Finally, the different learning formats of the MOOC have been made available to other German medical faculties to support their integration into, for example, gaming applications or blended learning formats.

### Limitations

The low response rate of 19% for the knowledge MCQs and self-assessment questions and 15% for the course feedback based on 2061 MOOC learners resulted in limitations in the evaluation of our MOOC. However, the response rate in relation to the 473 users who completed the course was higher at 82% for the knowledge MCQs and self-assessment questions. With regard to the feedback providers, we can assume that they have also completed the course in full because the request to evaluate the course was made immediately after the exam had been completed. In relation to these 473 learners, the response rate for the course feedback is then 64%. However, as the feedback survey was anonymous, we were unable to check whether the feedback responders had actually completed the course. We observed a significant increase in knowledge and self-confidence among the 389 interested learners who answered the knowledge MCQs and self-assessment questions at the beginning and end of the course. We could not exclude the possibility that MOOC learners might use other additional sources to answer the MCQs correctly. Moreover, we received positive course feedback from learners who stayed until the end of the MOOC. Unfortunately, we lacked the evaluation of those who did not complete the MOOC, which could have inflated the increase in knowledge and the positive nature of the course feedback.

## Conclusion

Overall, the MOOC was rated very positively by course participants. MOOC learners who completed the knowledge MCQs and self-assessment questions at the beginning and the end of the MOOC achieved an impressive increase in knowledge and self-confidence in the prudent use of antibiotics. By making all our MOOC content and learning formats available for free, we invite medical faculties in German-speaking countries to incorporate them into their undergraduate education programs. In addition, the MOOC and its individual elements may offer excellent opportunities for linking digital and traditional teaching at medical schools, for instance as blended learning. Integrating MOOCs on other topics into student teaching could also be valuable. Furthermore, the establishment of personalized learning paths could be considered for creating greater incentives to successfully complete the MOOC.

## Supplementary Information

Below is the link to the electronic supplementary material.

Supplementary Material 1. Structure of the MOOC and course content.

Supplementary Material 2. User behaviour and learning success of MOOC participants and their course feedback.

## Data Availability

The datasets used and analysed during the current study are available from the corresponding author on reasonable request.

## Abbreviations

ABS: antibiotic stewardship
AMR: antimicrobial resistance
CAP: community-acquired pneumonia
HPI: Hasso-Plattner-Institute
IQR: interquartile range
KF: key feature
M: module
MCQ: multiple choice question
MOOC: massive open online course
n: count
NKLM: National Competence-Based Catalogue of Learning Objectives for Undergraduate Medical Education
RAI: responsible antibiotic use via information and communication (project name)
