# Supplementary material for: Development and evaluation of a massive open online course (MOOC) to teach medical students the prudent use of antibiotics
Source: Antimicrob Resist Infect Control. 2025 Sep 22;14:105. doi: 10.1186/s13756-025-01640-4 (PMC12455833; doi:10.1186/s13756-025-01640-4)
Supplement: Supplementary file 1 — Supplementary Material 1. Structure of the MOOC and course content. [file 13756_2025_1640_MOESM1_ESM.pdf]

Electronic supplementary material

Additional file 1

“Development and evaluation of a massive open online course (MOOC) to teach medical students the prudent use of antibiotics”
Wiese-Posselt, M et al.

Structure of the MOOC and course content

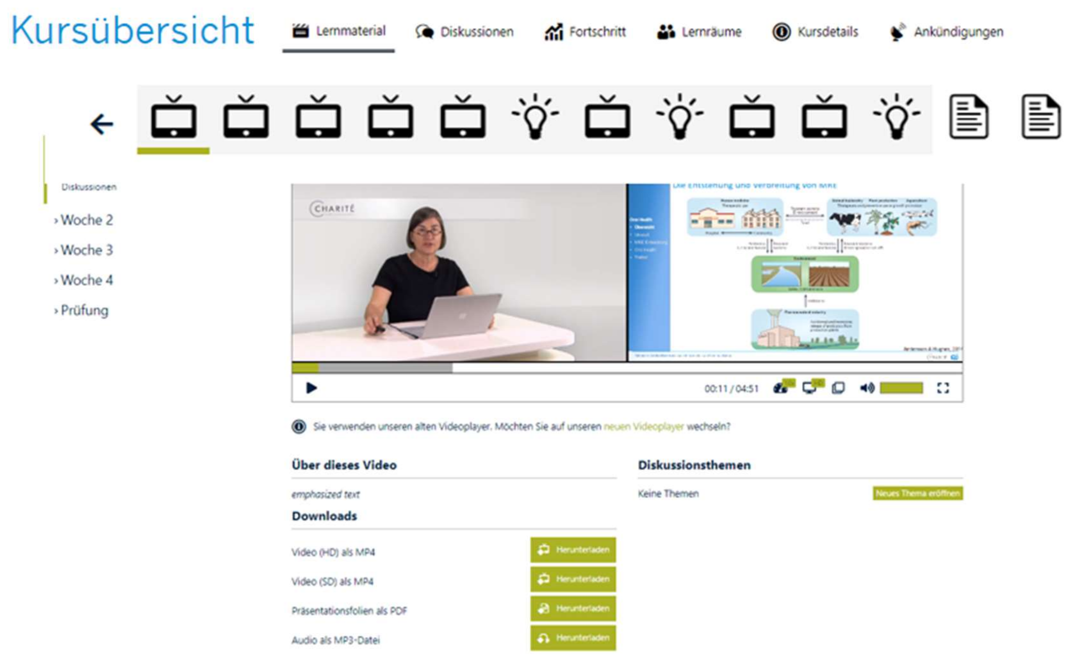

This is a visualisation of the basic structure of the MOOC (the screen is originally from the HPI platform). On the left, you can select the module, and the symbols are depicted at the top. The screen symbol represents a video and the light bulb represents a self-test. The sheet represents additional reading material.

During the course, we also integrated learning units from our other RAI students project learning formats (explanatory films, podcast episodes and interactive patient cases). These were assigned their own symbols. In the subsequent content listing, main items are written in black and supporting items in grey.

Note: *Some podcast episodes and videos of lecturers were only added to the MOOC after the study phase had finished, i.e. in October 2022. The corresponding podcast episodes and learning videos are marked in italics below.*

The podcast InfectED is available on:

- Podigee: https://rai-infected.podigee.io/
- Spotify: https://open.spotify.com/show/08qV6dw4to2pl4Yri2wGL2
- Apple Podcasts: https://podcasts.apple.com/us/podcast/infected-der-antibiotika-podcast/id1601400216

Content of the MOOC

First: General information

- General information about the MOOC
- Introduction of the lecturers
- Announcements during the course
- Discussions blog

Module 1

- Video of a lecturer: Introduction
- Self-assessment before the start of the course (5 questions)
- Introductory questions (16 multiple-choice questions (MCQs))
- Supplementary explanatory film: 'Structure of a bacterium' (https://www.youtube.com/watch?v=1UwMLDzTAtg)
- Video of a lecturer: Bacteriology I
- Video of a lecturer: Bacteriology II
- Bacteriology learning check (8 MCQs)
- Video of a lecturer: Microbiological diagnostics
- Video of a lecturer: Preanalytics
- Video of a lecturer: Interpretation of bacteriological findings
- Learning check: Microbiological diagnostics (9 MCQs)
- *(Since October 2022: Related podcast episode: #6 Blood, urine and pus: Microbiology diagnostics* (https://rai-infected.podigee.io/8-mibi))
- List of further links to Module 1 (see reference list below)

Module 2

- Supplementary explanatory film: 'Mechanisms of action of antibiotics' (https://www.youtube.com/watch?v=bhTQmGzl82w)
- Video of a lecturer: Mechanisms of action of antibiotics; antibiotic classes
- Video of a lecturer: Spectra of action of antibiotics I: Beta-lactam antibiotics
- Video of a lecturer: Activity spectra of antibiotics II: Non-beta-lactam antibiotics
- Learning check: Modes of action of antibiotics (5 MCQs)
- Video of a lecturer: Pharmacology of antibiotics
- Video of a lecturer: Undesirable effects of antibiotics

- Pharmacology and adverse effects of antibiotics learning check (MCQs)
- Supplementary explanatory film: 'Microbiome and antibiotic-associated diarrhoea' (https://www.youtube.com/watch?v=buQ9FVqYosw)
- Related podcast episodes:
  - #3 What we can learn from the Bedouins: *Clostridioides difficile* infections (https://rai-infected.podigee.io/4-cdi)
  - *(Since October 2022: #12: PharmakoLogisch! Pharmacology in antibiotic therapy* (https://rai-infected.podigee.io/15-pharma))
- List of further links to Module 2 (see reference list below)

Part 1 of the exam:

- Information on the exam
- Exam part 1 covers the contents of Modules 1 and 2 and consists of 40 MCQs.

Module 3

- Supplementary explanatory films: 'Selection and resistance mechanisms' (https://www.youtube.com/watch?v=9gk061UEvdw) and 'Resistance transmission and transmission' (https://www.youtube.com/watch?v=0hERDwLM_Pc)
- Video of a lecturer: Selection and transmission
- Supplementary explanatory film: 'Multi-resistant pathogens' (https://www.youtube.com/watch?v=8K89gyuYaiI)
- Video of a lecturer: Multi-resistant pathogens (MDRO)
- Video of a lecturer: Epidemiology of multidrug-resistant pathogens
- Learning check: Selection, transmission and MDRO (14 MCQs)
- Supplementary explanatory film: 'Appropriate antibiotic therapy' (https://www.youtube.com/watch?v=51NMrFrqV7k)
- Video of a lecturer: Principles of an appropriate antibiotic therapy
- Video of a lecturer: One Health
- Learning check: Appropriate antibiotic therapy and One Health (7 MCQs)
- *Related podcast episodes, since October 2022: #5 – Primum non nocere! Infection and transmission prevention in hospitals* (https://rai-infected.podigee.io/6-ipc) , *#13 Thinking outside the box: Antibiotic resistance and One Health* (https://rai-infected.podigee.io/14-vet)).
- List of further links to Module 3 (see reference list below)

Module 4

- Video of a lecturer: Prudent antibiotic therapy in general medicine: Bronchitis, sinusitis, COPD exacerbation and pneumonia
- *Video of a lecturer: Antibiotic Stewardship in Paediatrics, Community-Acquired Pneumonia in Paediatrics - The video was uploaded in October 2022 and was not included in the evaluation of the MOOC in the period from July 1, 2021 to June 30, 2022.*
- Learning check: General Medicine
- Interactive patient cases as a supplement: Use of the Casetrain software (University of Würzburg, Germany; https://casetrain.uni-wuerzburg.de/):
  - 'Cough in February'

- ‘A woman in autumn’
- 'Connected and coughing up'
- 'Patricia just won't listen'
- 'Her throat hurts'

If you would like to access the interactive Casetrain cases, please contact us: rai-info@charite.de.

- Video of a lecturer: Prudent antibiotic therapy in internal medicine: Bacterial meningitis, endocarditis and *Staphylococcus aureus* bacteraemia
- Learning check: Internal medicine (7 MCQs)
- Video of a lecturer: Urinary tract infections
- Learning check: Urinary tract infections (10 MCQs)
- Video of a lecturer: Perioperative antibiotic prophylaxis in surgery
- Learning check: Perioperative antibiotic prophylaxis in surgery
- Interactive patient cases as a supplement: Use of the Casetrain software (University of Würzburg, Germany; https://casetrain.uni-wuerzburg.de/):
    - 'Headache and fever'
    - 'A sporty patient with a feverish handicap'
    - 'Patient with a painfully reddened leg'
    - 'It burns!'

If you would like to access the interactive Casetrain cases, please contact us: rai-info@charite.de.

- *Video of a lecturer: Abdominal infections - The video was uploaded in autumn 2022 and was not included in the evaluation of the MOOC in the period from July 1, 2021 to June 30, 2022.*
- Video of a lecturer: Intensive care medicine: Sepsis and pneumonia (CAP and VAP)
- Learning check: Intensive care medicine (8 MCQs)
- Interactive patient cases as a supplement: Use of the Casetrain software (University of Würzburg, Germany; https://casetrain.uni-wuerzburg.de/):
    - 'He who has nothing is given'
    - 'Suddenly this cough'

If you would like to access the interactive Casetrain cases, please contact us: rai-info@charite.de.

- Related podcast episodes:
    - #1 From the GP to the ICU: Community-acquired pneumonia (https://rai-infected.podigee.io/2-cap)
    - #2 Is that really a urinary tract infection? *Staphylococcus aureus* bacteraemia (https://rai-infected.podigee.io/3-sab)
    - #4 Of slacklines and other dangers: Skin and soft tissue infections (https://rai-infected.podigee.io/5-ssti)

    Since October 2022:

    - *#8 PAPperlapapp: Perioperative antibiotic prophylaxis* (https://rai-infected.podigee.io/10-pap)
    - *#9 Rest, ibuprofen or an antibiotic after all? Acute tonsillitis and acute otitis media in paediatric practice* (https://rai-infected.podigee.io/11-paed)
    - *#11 Morbus Marlboro: Exacerbation of COPD due to infection* (https://rai-infected.podigee.io/13-copd)
    - *#14 What did Gustav Mahler die of? Infective endocarditis* (https://rai-infected.podigee.io/16-endokarditis)
    - *#15A It burns! Urinary tract infections with complicating factors* (https://rai-infected.podigee.io/17-hwi1)

- *#15B ORENUC: Urinary tract infections in the hospital* (https://rai-infected.podigee.io/18-hwi2)
- *#16 Drawers and the in-between: Nosocomial pneumonia* (https://rai-infected.podigee.io/19-hap)
- *#17 Sicker than ever! Sepsis* (https://rai-infected.podigee.io/20-sepsis)

- List of further links to Module 4 (see reference list below)

Part 2 of the exam:

- Information on the exam
- Exam part 2 covers the contents of Modules 3 and 4 and consists of 40 MCQs.

Self-assessment at the end of the MOOC.

Course Feedback: Forwarding to another website (LimeSurvey https://www.limesurvey.org/de) to complete an anonymous questionnaire evaluating the MOOC.

Certificate: "Antibiotic licence"; it is available for download if you pass the exam, i.e. if you correctly answer at least 60 out of 80 MCQs.

Note: Power point presentation of all lecturer videos can be provided via rai-info@charite.de

List of references of the content of the MOOC

Last accessed on the websites on June 2, 2025. The list also includes the topics 'ABS in paediatrics' and 'abdominal infections'.

Module 1: Further information

Guide from the Robert-Koch Institute, Berlin:
https://www.rki.de/DE/Aktuelles/Publikationen/RKI-Ratgeber/rki-ratgeber-node.html

Systematics of bacteria; from online learning tool for medical students (AMBOSS):
https://www.amboss.com/de/wissens-hub/systematik-der-bakterien

Training film on taking blood cultures:
https://www.doccheck.com/de/detail/videos/3756-schulungsvideos-zur-praeanalytik-blutkulturen

Module 2: Further information

WHO: critical important antimicrobials:
https://iris.who.int/bitstream/handle/10665/325036/WHO-NMH-FOS-FZD-19.1-eng.pdf?isAllowed=y&sequence=1

https://iris.who.int/bitstream/handle/10665/312266/9789241515528-eng.pdf

Antibiotics; from online learning tool for medical students (AMBOSS):
https://www.amboss.com/de/wissens-hub/antibiotika-mosaik

University Heidelberg, Department of Clinical Pharmacology & Pharmacoepidemiology University Hospital
https://www.dosing.de/

Charité – Universitätsmedizin Berlin, Institute of Clinical Pharmacology and Toxicology
https://www.embryotox.de/arzneimittel/

Federal Institute for Drugs and Medical Devices (BfArM), urgent safety message 2018:
https://www.akdae.de/fileadmin/user_upload/akdae/Arzneimittelsicherheit/RHB/Archiv/2018/20181026.pdf

Federal Institute for Drugs and Medical Devices (BfArM), urgent safety message 2019:
https://www.akdae.de/fileadmin/user_upload/akdae/Arzneimittelsicherheit/RHB/Archiv/2019/20190408.pdf

Module 3: Further information

Understanding the mechanisms and drivers of antimicrobial resistance, The Lancet 2016:
https://www.sciencedirect.com/science/article/pii/S0140673615004730?via=ihub

Surveillance-Atlas of the European Centre for Disease Prevention and Control (ECDC):
http://atlas.ecdc.europa.eu/public/index.aspx

Interactive database of the RKI's antibiotic resistance surveillance project (ARS):
https://ars.rki.de/Content/Database/ResistanceOverview.aspx

Brochures on the One Health concept and on selection and resistance mechanisms from the RAI project:
https://www.rai-projekt.de/tiermedizin

Module 4: Further information

Petruschke I, Pletz MW, Bleidorn J. Infektionen der oberen Atemwege. CME 12/2019:
https://www.springermedizin.de/pharyngitis/bronchitis/infektionen-der-oberen-atemwege/17496238

Laxminarayan R, Zulfiqar AB. Antimicrobial resistance—a threat to neonate survival. Lancet Global Health, 2016:
https://www.thelancet.com/journals/langlo/article/PIIS2214-109X%2816%2930221-2/fulltext

German Society for Infectiology: Infektiopedia:
https://infektiopedia.de/wiki/Hauptseite

Hagel S, Kaasch AJ, Weis S, Seifert H, Pletz MW, Rieg S. *Staphylococcus aureus* Blutstrominfektion – eine interdisziplinäre Herausforderung. Anästhesiol Intensivmed Notfallmed Schmerzther 2019: https://www.thieme-connect.com/products/ejournals/pdf/10.1055/a-0756-8263.pdf

Gágyor I, Bleidorn J, Kochen MM, Schmiemann G, Wegscheider K, Hummers-Pradier E. Ibuprofen versus fosfomycin for uncomplicated urinary tract infection in women: randomised controlled trial. *BMJ* 2015*;* 351 doi: https://doi.org/10.1136/bmj.h6544
https://www.ncbi.nlm.nih.gov/pmc/articles/PMC4688879/

Kaußner Y, Röver C, Heinz J, Hummers E, Debray TPA, Hay AD, Heytens S, Vik I, Little P, Moore M, Stuart B, Wagenlehner F, Kronenberg A, Ferry S, Monsen T, Lindbak M, Friede T, Gágyor I. Reducing antibiotic use in uncomplicated urinary tract infections in adult women: a systematic review and individual participant data meta-analysis. CMI 2022: https://www.sciencedirect.com/science/article/pii/S1198743X22003305?via%3Dihub

Dreger NM, Degener S, Ahmad-Nejad P, Wöbker G, Roth S. Urosepsis – etiology, diagnosis and treatment. Dtsch Ärztbl Int 2015: https://www.aerzteblatt.de/archiv/173138/Urosepsis-Ursache-Diagnose-und-Therapie

Reference data on the pathogen spectrum for wound infections in defined operations of the German National Reference Center for Surveillance of Nosocomial Infections (KISS): https://www.nrz-hygiene.de/KISS-Modul/referenzdaten/KISS/OP

National information on sepsis: https://sepsisakademie.de/

Reference data on the pathogen spectrum of defined nosocomial infections in intensive care units of the German National Reference Center for Surveillance of Nosocomial Infections (KISS): https://www.nrz-hygiene.de/KISS-Modul/referenzdaten/KISS/ITS-KISS-Erreger-Surveillance

Online journal on infectious diseases: https://www.infektio.de/

Further training in infection medicine for clinical colleagues, Berlin: https://www.sjk.de/kliniken/klinik-infektiologie/infektoskop-ein-streifzug-durch-die-infektiologie.html

Guidelines:

In Germany, evidence-based guidelines are developed by the medical societies. This process, as well as the publication and provision of the guidelines, is carried out by the German Association of Scientific Medical Societies (Arbeitsgemeinschaft der Wissenschaftlichen Medizinischen Fachgesellschaften e.V., https://www.awmf.org/).

The lecturers' learning videos were based on the following guidelines:

- https://register.awmf.org/de/leitlinien/detail/020-003
- https://register.awmf.org/de/leitlinien/detail/053-013
- https://register.awmf.org/de/leitlinien/detail/053-012
- https://register.awmf.org/de/leitlinien/detail/nvl-003
- https://register.awmf.org/de/leitlinien/detail/020-020
- https://register.awmf.org/assets/guidelines/048-015l_S2k_Antibiotic-Stewardship-ABS-Konzeption-Umsetzung-stationaere-Kinder-Jugendmedizin_2019-06.pdf
- https://register.awmf.org/de/leitlinien/detail/048-013
- https://register.awmf.org/de/leitlinien/detail/030-089

- https://register.awmf.org/de/leitlinien/detail/043-044
- https://euprevent.eu/wp-content/uploads/2017/01/PEG-perioperative-Prophylaxe-2010.pdf
- https://register.awmf.org/de/leitlinien/detail/067-009
- https://register.awmf.org/de/leitlinien/detail/079-001
- https://register.awmf.org/de/leitlinien/detail/020-013
- https://register.awmf.org/de/leitlinien/detail/082-006
- https://register.awmf.org/de/leitlinien/detail/021-017

The following international guidelines were also considered:

- https://www.escardio.org/Guidelines/Clinical-Practice-Guidelines/Endocarditis-Guidelines
- https://www.idsociety.org/globalassets/idsa/practice-guidelines/infective-endocarditis-in-adults-diagnosis-antimicrobial-therapy-and-management-of-complications.pdf
- https://journals.lww.com/ccmjournal/fulltext/2021/11000/surviving_sepsis_campaign__international.21.aspx
- https://www.sciencedirect.com/science/article/pii/S2352556815000582?via%3Dihub
