# Supplementary material for: Development and evaluation of a massive open online course (MOOC) to teach medical students the prudent use of antibiotics
Source: Antimicrob Resist Infect Control. 2025 Sep 22;14:105. doi: 10.1186/s13756-025-01640-4 (PMC12455833; doi:10.1186/s13756-025-01640-4)
Supplement: Supplementary file 2 — Supplementary Material 2. User behaviour and learning success of MOOC participants and their course feedback. [file 13756_2025_1640_MOESM2_ESM.docx]

**Electronic supplementary material**

**Additional file 2**

**“Development and evaluation of a massive open online course (MOOC) to teach medical students the prudent use of antibiotics”**

**Wiese-Posselt, M et al.**

**I.)** **User behaviour and learning success of MOOC participants**

**Table S1:** Information on MOOC participants differentiated by completeness of response to the 16 knowledge and 5 self-assessment questions; MOOC term 2021-2022.

|  | **Responses complete,  n = 389** | **Responses incomplete,  n = 1,672** | **p-value^a^** |
| --- | --- | --- | --- |
| **Country of registration [n (%)]** |  |  | 0.4 |
| - Germany | 343 (89%) | 1,470 (88%) |  |
| - Switzerland | 12 (3.1%) | 35 (2.1%) |  |
| - Austria | 7 (1.8%) | 51 (3.1%) |  |
| - Other | 25 (6.5%) | 111 (6.7%) |  |
| - Unknown | 2 | 5 |  |
| **Session duration [min](^b^)** | 28 (0;21;41;110) | 9 (0;1;21;180) | <0.001 |
| - Of those, unknown | 0 | 6 |  |
| **Video play activity [n](^b^)** | 11 (0;0;21;39) | 0 (0;0;2;22) | <0.001 |
| - Of those, unknown | 0 | 6 |  |
| **Learning items visited: [^c^](^b^)** |  |  |  |
| - Module 1 (M1) | 1.00 (0.18;1.00;1.00;1.00) | 0.45 (0.00;0.09;0.82;1.00) | <0.001 |
| - Module 2 (M2) | 0.89 (0.00;0.89;1.00;1.00) | 0.00 (0.00;0.00;0.33;1.00) | <0.001 |
| - Module 3 (M3) | 1.00 (0.00;1.00;1.00;1.00) | 0.00 (0.00;0.00;0.00;1.00) | <0.001 |
| - Module 4 (M4) | 0.92 (0.00;0.83;1.00;1.00) | 0.00 (0.00;0.00;0.00;1.00) | <0.001 |
| **Participation in self-tests: [^c^](^b^)** |  |  |  |
| - M1 bacteriology | 1.00 (0.38;0.88;1.00;1.00) | 1.00 (0.13;0.88;1.00;1.00) | 0.013 |
| Of those, unknown [n] | 45 | 1,190 |  |
| - M1 microbiological diagnostics | 0.89 (0.56;0.89;1.00;1.00) | 0.89 (0.00;0.78;1.00;1.00) | 0.004 |
| Of those, unknown [n] | 50 | 1,322 |  |
| - M2 antibiotics: mechanisms, spectra | 0.89 (0.22;0.78;1.00;1.00) | 0.78 (0.11;0.67;0.94;1.00) | <0.001 |
| Of those, unknown [n] | 57 | 1,436 |  |
| - M2 pharmacology, side-effects | 0.86 (0.21;0.79;0.93;1.00) | 0.86 (0.00;0.71;0.93;1.00) | 0.009 |
| Of those, unknown [n] | 59 | 1,513 |  |
| - M3 selection, transmission | 0.93 (0.00;0.86;1.00;1.00) | 0.93 (0.00;0.79;1.00;1.00) | 0.045 |
| Of those, unknown [n] | 56 | 1,576 |  |
| - M3 antibiotic therapy, One Health | 1.00 (0.00;1.00;1.00;1.00) | 1.00 (0.00;1.00;1.00;1.00) | 0.2 |
| Of those, unknown [n] | 54 | 1,587 |  |
| - M4 general medicine | 0.91 (0.00;0.91;1.00;1.00) | 0.91 (0.00;0.82;1.00;1.00) | 0.018 |
| Of those, unknown [n] | 52 | 1,584 |  |
| - M4 internal medicine | 0.86 (0.00;0.71;1.00;1.00) | 0.86 (0.00;0.71;1.00;1.00) | 0.7 |
| Of those, unknown [n] | 57 | 1,596 |  |
| - M4 urinary tract infections | 1.00 (0.00;0.90;1.00;1.00) | 1.00 (0.00;0.90;1.00;1.00) | 0.6 |
| Of those, unknown [n] | 58 | 1,602 |  |
| - M4 surgery | 1.00 (0.00;0.83;1.00;1.00) | 1.00 (0.00;0.83;1.00;1.00) | 0.8 |
| Of those, unknown [n] | 64 | 1,604 |  |
| - M4 intensive care medicine | 0.86 (0.00;0.71;1.00;1.00) | 0.86 (0.00;0.71;1.00;1.00) | 0.2 |
| Of those, unknown [n] | 66 | 1,604 |  |
| **Discussion forum activity [n](^b^)** | 5.0 (0.0;2.0;9.0;73.0) | 0.0 (0.0;0.0;1.0;29.0) | <0.001 |

^a^ Wilcoxon rank-sum test; Pearson's Chi-squared test

^b^ Median (minimum;25%;75%;maximum)

^c^ Proportional frequency

**Table S2:** Overview of the 16 knowledge MCQs and the answers given by the 389 learners at the beginning and end of the MOOC. The increase in correct answers when answering the questions at the end of the MOOC was significant for all questions (McNemar test; p-value < 0.001). MOOC term 2021-2022.

^a^ Given in number and percentage n (%)

**Correct answers** are written in bold and marked with **(c.a.)**

**Table S2.01**: Which bacteria are not Gram-positive?

| Possible answers | Answers at the beginning of the MOOC^a^ | Answers at the end of the MOOC^a^ |
| --- | --- | --- |
| Clostridia | 96 (25%) | 7 (1.8%) |
| Enterococci | 57 (15%) | 10 (2.6%) |
| **Neisseria (c.a.)** | **217 (56%)** | **371 (95%)** |
| Staphylococci | 10 (2.6%) | 1 (0.3%) |
| Streptococci | 9 (2.3%) | 0 (0%) |

**Table S2.02:** Which of the following pathogens does not belong to the enterobacteriaceae?

| Possible answers | Answers at the beginning of the MOOC^a^ | Answers at the end of the MOOC^a^ |
| --- | --- | --- |
| *Escherichia coli* | 6 (1.5%) | 0 (0%) |
| *Klebsiella pneumoniae* | 69 (18%) | 5 (1.3%) |
| *Proteus mirabilis* | 17 (4.4%) | 8 (2.1%) |
| ***Pseudomonas aeruginosa* (c.a.)** | **251 (65%)** | **363 (93%)** |
| *Serratia marcescens* | 46 (12%) | 1. 3.3%) |

**Table S2.03:** Which is not part of the preanalytics?

| Possible answers | Answers at the beginning of the MOOC^a^ | Answers at the end of the MOOC^a^ |
| --- | --- | --- |
| Selection of the suitable sample material | 3 (0.8%) | 0 (0%) |
| Appropriate storage conditions for the sample after collection | 8 (2.1%) | 2 (0.5%) |
| Indication for diagnostics | 13 (3.3%) | 0 (0%) |
| **Interpretation of resistance testing (c.a.)** | **357 (92%)** | **385 (99%)** |
| Clinical information for the laboratory | 8 (2.1%) | 2 (0.5%) |

**Table S2.04:** Which antibiotic belongs to the beta-lactam antibiotics?

| Possible answers | Answers at the beginning of the MOOC^a^ | Answers at the end of the MOOC^a^ |
| --- | --- | --- |
| Cephalosporins, makrolides, penicillins | 35 (9.0%) | 0 (0%) |
| Fluorchinolons, carbapenems, penicillins | 9 (2.3%) | 0 (0%) |
| Makrolides, fluorchinolons, glycopeptides | 8 (2.1%) | 2 (0.5%) |
| **Penicillins, carbapenems, cephalosporins (c.a.)** | **291 (75%)** | **385 (99%)** |
| Penicillins, makrolides, beta-lactamase inhibitors | 46 (12%) | 2 (0.5%) |

**Table S2.05:** How do fluoroquinolones work?

| Possible answers | Answers at the beginning of the MOOC^a^ | Answers at the end of the MOOC^a^ |
| --- | --- | --- |
| Insertion of channels into the cell wall that lead to lysis | 15 (3.9%) | 0 (0%) |
| Inhibition of bacterial protein synthesis | 53 (14%) | 6 (1.5%) |
| Inhibition of folic acid synthesis | 43 (11%) | 3 (0.8%) |
| Disruption of bacterial cell wall synthesis | 27 (6.9%) | 1 (0.3%) |
| **Interruption of bacterial DNA replication by inhibition of gyrase (c.a.)** | **251 (65%)** | **379 (97%)** |

**Table S2.06:** Which of the following agents is the first-line treatment for proven methicillin-sensitive *Staphylococcus aureus*?

| Possible answers | Answers at the beginning of the MOOC^a^ | Answers at the end of the MOOC^a^ |
| --- | --- | --- |
| Ceftriaxon | 55 (14%) | 3 (0.8%) |
| **Flucloxacillin (c.a.)** | **213 (55%)** | **375 (96%)** |
| Gentamicin | 17 (4.4%) | 3 (0.8%) |
| Meropenem | 22 (5.7%) | 0 (0%) |
| Piperacillin-tazobactam | 82 (21%) | 8 (2.1%) |

**Table S2.07:** Which statement best describes the selection of antibiotic resistance?

| Possible answers | Answers at the beginning of the MOOC^a^ | Answers at the end of the MOOC^a^ |
| --- | --- | --- |
| Uptake of free DNA by a bacterium | 3 (0.8%) | 1 (0.3%) |
| **Survival or reproductive advantages of bacteria with certain properties, e.g. antibiotic resistance under antibiotic treatment (c.a.)** | **324 (83%)** | **376 (97%)** |
| Transfer of DNA between bacteria by bacteriophages | 2 (0.5%) | 2 (0.5%) |
| Patient-to-patient transmission of pathogens | 0 (0%) | 4 (1.0%) |
| Transfer of resistance genes between bacteria by plasmid exchange via sex pili | 60 (15%) | 6 (1.5%) |

**Table S2.08:** Which abbreviation is correct (note: abbreviations used in Germany)?

| Possible answers | Answers at the beginning of the MOOC^a^ | Answers at the end of the MOOC^a^ |
| --- | --- | --- |
| MRE: multiresistant enterococci | 56 (14%) | 4 (1.0%) |
| **MRGN: multiresistant gram-negative pathogens (c.a.)** | **305 (78%)** | **382 (98%)** |
| MRSA: methicillin-sensitive *Staphylococcus aureus* | 18 (4.6%) | 2 (0.5%) |
| MRSA: multiresistant rod-shaped bacteria | 3 (0.8%) | 0 (0%) |
| VRE: penicillin V-resistant enterobacterales | 7 (1.8%) | 1 (0.3%) |

**Table S2.09:** The approximate percentage of carbapenem-resistant *Klebsiella pneumoniae* strains in India is?

| Possible answers | Answers at the beginning of the MOOC^a^ | Answers at the end of the MOOC^a^ |
| --- | --- | --- |
| 1% | 5 (1.3%) | 2 (0.5%) |
| 10% | 49 (13%) | 4 (1.0%) |
| 30% | 190 (49%) | 21 (5.4%) |
| 5% | 21 (5.4%) | 1 (0.3%) |
| **50% (c.a.)** | **124 (32%)** | **361 (93%)** |

**Table S2.10:** Which statement about the principles of prudent antibiotic therapy is true?

| Possible answers | Answers at the beginning of the MOOC^a^ | Answers at the end of the MOOC^a^ |
| --- | --- | --- |
| The antibiotic pack should always be used up to avoid antibiotic resistance. | 55 (14%) | 1 (0.3%) |
| The duration of antibiotic therapy should be precisely determined at the beginning of treatment and should not be questioned during the course of treatment. | 5 (1.3%) | 1 (0.3%) |
| De-escalation of a calculated therapy usually takes place after 7 days. | 12 (3.1%) | 1 (0.3%) |
| A calculated antibiotic therapy should always consider gram-positive and gram-negative pathogens in the spectrum of action. | 39 (10%) | 7 (1.8%) |
| **The under dosing of antibiotics in particular favours the development of resistance. (c.a.)** | **278 (71%)** | **379 (97%)** |

**Table S2.11:** Acute bronchitis: What percentage of cases are viral?

| Possible answers | Answers at the beginning of the MOOC^a^ | Answers at the end of the MOOC^a^ |
| --- | --- | --- |
| 5% | 7 (1.8%) | 1 (0.3%) |
| **90% (c.a.)** | **216 (56%)** | **384 (99%)** |
| 30% | 35 (9.0%) | 1 (0.3%) |
| 50% | 20 (5.1%) | 1 (0.3%) |
| 70% | 111 (29%) | 2 (0.5%) |

**Table S2.12:** Acute bronchitis: Which of the following statements is correct?

| Possible answers | Answers at the beginning of the MOOC^a^ | Answers at the end of the MOOC^a^ |
| --- | --- | --- |
| **The cough can last up to 6 weeks. (c.a.)** | **149 (38%)** | **382 (98%)** |
| Acute bronchitis lasting longer than a week is usually an indication for antibiotic therapy. | 32 (8.2%) | 2 (0.5%) |
| Acute bronchitis is bacterial in about 30% of cases. | 70 (18%) | 2 (0.5%) |
| Yellow-green sputum is a good indicator of bacterial superinfection. | 129 (33%) | 2 (0.5%) |
| Pertussis does not play a role in the differential diagnosis of adults with acute bronchitis, as it is a childhood disease. | 9 (2.3%) | 1 (0.3%) |

**Table S2.13:** What are the most common pathogens in community-acquired pneumonia?

| Possible answers | Answers at the beginning of the MOOC^a^ | Answers at the end of the MOOC^a^ |
| --- | --- | --- |
| *Enterococcus faecium, Proteus mirabilis, Klebsiella pneumoniae* | 4 (1.0%) | 0 (0%) |
| *Legionella pneumophila, Staphylococcus aureus, Chlamydia trachomatis* | 4 (1.0%) | 0 (0%) |
| *Staphylococcus aureus, Escherichia coli, Acinetobacter baumannii* | 7 (1.8%) | 1 (0.3%) |
| ***Streptococcus pneumoniae, Haemophilus influenza, Mycoplasma pneumonia* (c.a.)** | **348 (89%)** | **386 (99%)** |
| *Streptococcus pneumonia, Streptococcus viridans, Streptococcus pyogenes* | 25 (6.4%) | 2 (0.5%) |

**Table S2.14:** Community-acquired pneumonia (CAP): which statement is true?

| Possible answers | Answers at the beginning of the MOOC^a^ | Answers at the end of the MOOC^a^ |
| --- | --- | --- |
| Even in the case of outpatient treatment of a CAP, microbiological pathogen diagnostics should always be carried out. | 138 (35%) | 32 (8.2%) |
| Even in older patients, CAP can usually be treated well on an outpatient basis. | 66 (17%) | 31 (8.0%) |
| In the case of fever and typical auscultation findings, imaging (e.g. X-ray) can be omitted to confirm the diagnosis. | 79 (20%) | 29 (7.5%) |
| The choice of a calculated antibiotic therapy depends on the colour of the sputum. | 6 (1.5%) | 4 (1.0%) |
| **The duration of antibiotic therapy for mild to moderate CAP is usually 5 days. (c.a.)** | **100 (26%)** | **293 (75%)** |

**Table S2.15:** Community-acquired pneumonia (CAP): which antibiotic is the first choice for mild CAP without comorbidities?

| Possible answers | Answers at the beginning of the MOOC^a^ | Answers at the end of the MOOC^a^ |
| --- | --- | --- |
| **Amoxicillin (c.a.)** | **283 (73%)** | **381 (98%)** |
| Ceftriaxon | 41 (11%) | 4 (1.0%) |
| Doxycyclin | 7 (1.8%) | 1 (0.3%) |
| Levofloxacin | 13 (3.3%) | 2 (0.5%) |
| Penicillin V | 45 (12%) | 1 (0.3%) |

**Table S2.16:** Which statement is correct?

| Possible answers | Answers at the beginning of the MOOC^a^ | Answers at the end of the MOOC^a^ |
| --- | --- | --- |
| In the case of uncomplicated cystitis, the microbiological diagnosis should be made before a targeted antibiotic therapy is initiated. | 16 (4.1%) | 7 (1.8%) |
| If uncomplicated community-acquired cystitis is suspected, midstream urine should always be collected for microbiological diagnosis before starting antibiotic therapy. | 63 (16%) | 10 (2.6%) |
| Ciprofloxacin is one of the drugs of first choice for uncomplicated community-acquired cystitis. | 24 (6.2%) | 7 (1.8%) |
| **Acute pyelonephritis in a young female patient without concomitant diseases is a clear indication for antibiotic therapy. (c.a.)** | **273 (70%)** | **362 (93%)** |
| Asymptomatic bacteriuria in female patients over 60 years of age should be treated with antibiotics. | 10 (2.6%) | 3 (0.8%) |

**II.) Course Feedback**

Feedback on the MOOC, n = 304 (15%) of the MOOC participants

**Table S3:** Feedback: Assessment of the impact of the MOOC, n = 304; MOOC term 2021-2022.

|  | True^a^ | Fairly true^a^ | Not really true^a^ | Not true^a^ |
| --- | --- | --- | --- | --- |
| What I learned in the MOOC goes beyond the content of my studies. (n = 302) | 56 (19%) | 123 (41%) | 91 (30%) | 32 (11%) |
| What I have learned in the MOOC expands my knowledge in relevant areas. (n = 300) | 190 (63%) | 101 (34%) | 8 (3%) | 1 (0%) |
| The MOOC has given me a better understanding of the link between antibiotic administration and the development of resistance.  (n = 302) | 161 (53%) | 119 (39%) | 18 (6%) | 4 (1%) |
| The MOOC has given me a better overview of antibiotic therapy problems and their solutions. (n = 302) | 159 (53%) | 124 (41%) | 17 (6%) | 2 (1%) |
| What I learned in the MOOC is very relevant for my future work in the clinic or in medical practice. (n = 303) | 268 (89%) | 30 (10%) | 4 (1%) | 1 (0%) |
| What I have learned in the course I can apply directly to clinical work within my studies or in my everyday work as a doctor.  (n = 303) | 205 (68%) | 81 (27%) | 16 (5%) | 1 (0%) |

^a^ Given in number and percentage n (%)
